# Gastric non-secreting neuroendocrine tumor and hypochlorhydria-related hypergastrinemia: a case report

**DOI:** 10.1186/1752-1947-7-53

**Published:** 2013-02-22

**Authors:** Marco Biolato, Sergio Alfieri, Gianluca Ianiro, Marco Pizzoferrato, Giovanni Gasbarrini

**Affiliations:** 1 Institute of Internal Medicine, Catholic University, ‘A. Gemelli’ Hospital, Rome, Italy; 2 Department of Digestive Surgery, Catholic University, ‘A. Gemelli’ Hospital, Rome, Italy; 3 Fondazione Ricerca in Medicina ONLUS, Galleria Falcone e Borsellino 2, Bologna 40123, Italy

**Keywords:** Carcinoid, Gastric polyp, Gastrin, Gastrinoma, Zollinger–Ellison syndrome

## Abstract

**Introduction:**

Zollinger–Ellison syndrome is characterized by recurrent peptic ulcers and diarrhea that result from gastrin-secreting neuroendocrine tumors of the gastrointestinal tract; nevertheless, severe hypergastrinemia may also have alternative pathogenetic explanations.

**Case presentation:**

A 61-year-old woman of Caucasian origin presented with a history of epigastric pain and early satiety, severe hypergastrinemia (approximately 2000 pg/mL) and a neuroendocrine polyp in the corpus of her stomach. Chronic atrophic gastritis and intestinal metaplasia was present, but she denied use of acid suppressant drugs and the results of tests for *Helicobacter pylori* as well as gastric parietal cell and intrinsic factor antibodies were negative. She underwent a radical gastric tangential resection. Six months later, serum gastrin was still elevated despite lack of recurrence of tumor.

**Conclusion:**

The clinical picture was suggestive for a hypochlorhydria-related hypergastrinemia with subsequent development of a non-secreting carcinoid. We suggest a periodic endoscopic follow-up in patients with severe hypochlorhydria-related hypergastrinemia in order to earlier detect neuroendocrine polyps.

## Introduction

Initially described in 1955 [1], Zollinger–Ellison syndrome is characterized by multiple and recurrent peptic ulcers and persistent diarrhea that result from gastrin-secreting neuroendocrine tumors (gastrinomas) of the gastrointestinal tract [2]. Most gastrinomas are found in the pancreas, duodenum, or lymph nodes near the head of the pancreas, but they have also been found in other sites, and surgical resection is the antitumor treatment of choice [3]. Although both hypergastrinemia and histological features of neuroendocrine tumor are criteria for the diagnosis of Zollinger–Ellison syndrome, these data may also have an alternative pathogenetic explanation.

## Case presentation

**Figure 1:**
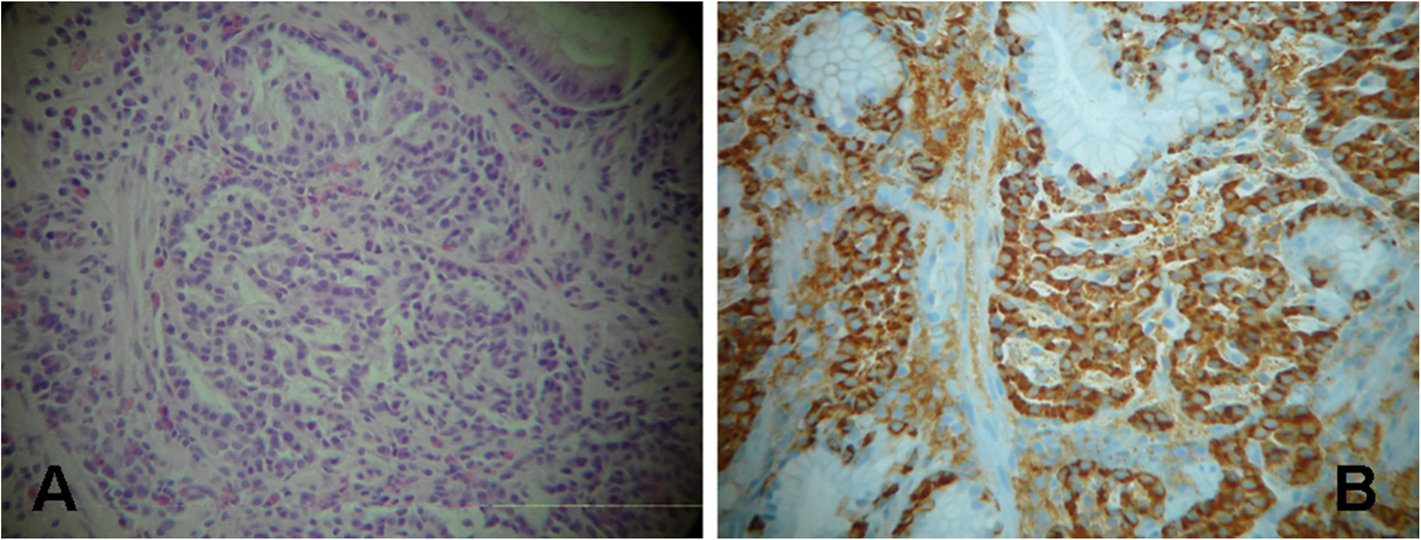
Hematoxylin and eosin staining (×100) of gastric biopsy showed trabecular structures of small round-oval cells (A); immunohistochemical staining (×100) showed that the cytoplasm is positive for synaptophysin, which confirms the diagnosis of neuroendocrine carcinoma (B).

**Figure 2:**
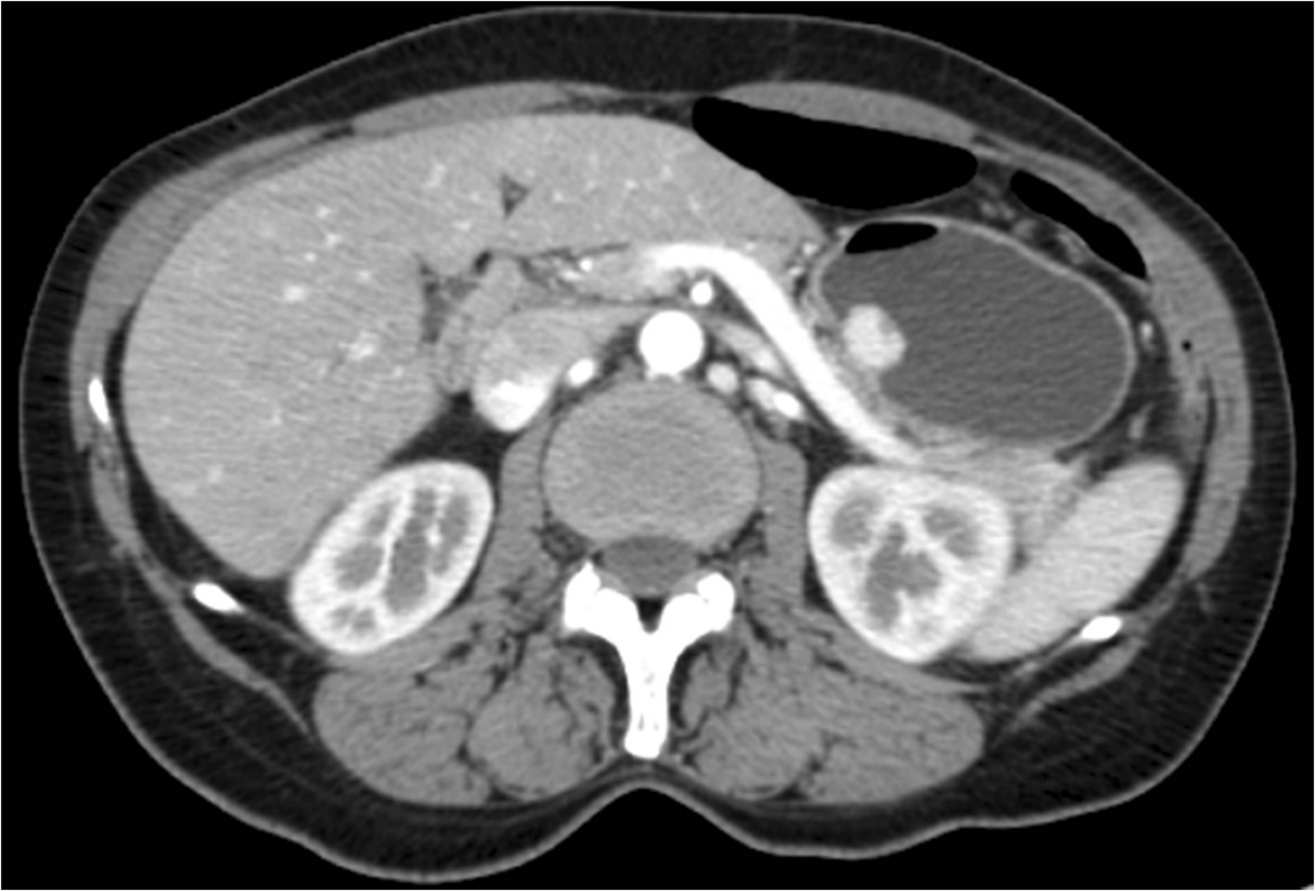
Abdomen computed tomography scan showed a gastric polyp with arterial enhancement and an apical area of necrosis.

**Figure 3:**
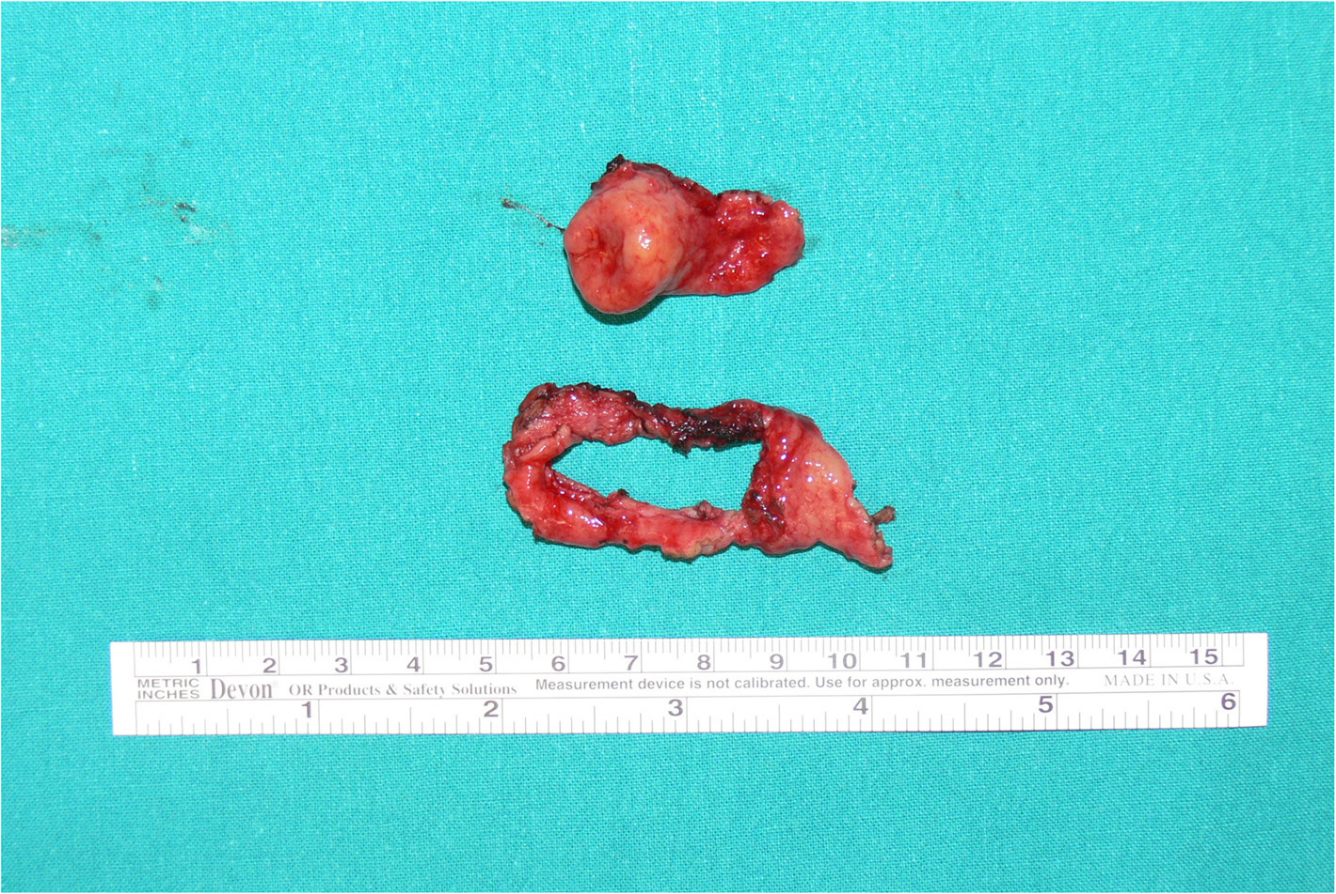
A gross photograph of the gastrectomy specimen showed a polyp (two cm in diameter) with an apical area of necrosis and the resected mucosal ring.

Here we report a case of a 61-year-old Italian woman of Caucasian origin who presented to our out-patient clinic with a three-month history of epigastric pain especially between meals, heartburn, early satiety and dyspepsia. The patient had a history of chronic lymphocytic thyroiditis, osteoporosis and small intestinal bacterial overgrowth. Her current medications included rifaximin, metronidazole and aluminum-magnesium hydroxide. The physical examination revealed a normal nutritional state. Laboratory tests revealed: serum gastrin 1900 pg/mL (reference range, 30–115), hemoglobin 11.6 g/dL (reference range, 12–16 in women), vitamin B12 level 139 pg/mL (reference range, 158–600) and internationalized normalized ratio 1.34 (reference range 0.8–1.2); because of this, she started phytonadione 10 mg/day. The results of other laboratory tests, including the white-cell count and the differential count, erythrocyte sedimentation rate, serum levels of electrolytes, glucose, total protein, albumin, globulin, cholesterol, lipids, folate, ferritin, amylase, lipase, renal function, liver function, thyroid function, serum neuron-specific enolase, chromogranin A, 5-hydroxy-indoloacetic acid, carcinoembryonic antigen, cancer antigen (CA) 19–9, CA 125, and gastric parietal cell and intrinsic factor antibodies, were normal. A urea breath test for *Helicobacter pylori* infection was negative. An upper gastrointestinal endoscopy was performed. A two-cm single sessile protruding-type polyp was found in the greater curvature of the patient’s stomach. Microscopic examination showed clumps of small round-oval shaped cells with areas arranged in a trabecular or solid pattern in the gastric mucosa (Figure 1). There were no area of necrosis and the KI-67 index was 3%. Immunohistochemical staining showed that the cancer cells were positive for neuron-specific enolase, cytokeratin and synaptophysin. The findings were those of a well-differentiated neuroendocrine carcinoma (carcinoid tumor). Mild chronic atrophic gastritis and intestinal metaplasia were present in the surrounding, non-neoplastic tissue in the corpus of her stomach. Thiazine staining for *H*. *pylori* was negative. Total body computed tomography showed no evidence of lymph node or hepatic metastases and confirmed a hypervascular 1.7×1.3 cm polyp in the stomach (Figure 2). The patient underwent a gastric tangential resection, restricted to the portion of corpus of gastric wall involved by tumor and sparing almost all of the stomach (Figure 3). Microscopic examination confirmed a well-differentiated, low-grade, neuroendocrine tumor of the stomach infiltrating the submucosal layer with microvascular invasion. The margin of the resection was free of disease. Postoperative staging was pT1 according to the Union for International Cancer Control’s TNM Classification of Malignant Tumours (7th edition). The patient’s postoperative course was uneventful and she remained in a good clinical condition. Six months later, her serum gastrin was 2011 pg/mL. A total body computed tomography scan and an upper gastrointestinal endoscopy excluded recurrence of the disease. A gastric antral biopsy showed mild chronic atrophic gastritis and intestinal metaplasia. Staining for *H*. *pylori* was negative. Immunohistochemical staining for cytokeratin and synaptophysin showed mild hyperplasia of neuroendocrine gastric cells. No tumor recurrence was revealed.

## Discussion

A severe (>1000 pg/mL) elevation of fasting serum gastrin concentration is usually suggestive for Zollinger–Ellison syndrome; on top of this, the use of acid suppressant medication (both proton pump inhibitors or H2-receptor antagonists), the presence of *H*. *pylori* infection and autoimmune achlorhydric atrophic gastritis without or with pernicious anemia may lead to mild-to-moderate hypergastrinemia [4,5]. In our case, the absence of symptoms of classical gastrinoma and the persistence of extremely elevated gastrin after radical tumor excision rule out the diagnosis of Zollinger–Ellison syndrome. The gastric biopsy performed six months after tumor resection showed chronic atrophic gastritis and intestinal metaplasia, with mild hyperplasia of antral G cells; this data, together with vitamin B12 deficiency anemia, suggest a hypochlorhydria-related hypergastrinemia. Although gastric parietal cell and intrinsic factor antibodies were both negative, cases of autoantibody-negative type A gastritis have been described [6]. More than 100 cases of gastric carcinoids in patients with pernicious anemia are described in the literature. Because the hormone gastrin regulates several important cellular processes in the gastric epithelium including proliferation, apoptosis, migration, invasion, tissue remodeling and angiogenesis [7], we hypothesize that the unusual and extremely elevated levels of gastrin facilitated the occurrence of gastric neuroendocrine tumor in this patient [8]. Some authors have suggested that small multiple gastric carcinoids associated with atrophic gastritis are indolent, despite patients having continuous hypergastrinemia [9,10]; notwithstanding, a surgical approach in tumors larger than two cm seems to be cautious.

## Conclusions

We suggest a periodic endoscopic follow-up in patients with severe (>1000 pg/mL) hypochlorhydria-related hypergastrinemia in order to earlier detect neuroendocrine polyps.

## Consent

Written informed consent was obtained from the patient for publication of this case report and accompanying images. A copy of the written consent is available for review by the Editor-in-Chief of this journal.

## Competing interests

The authors declare that they have no competing interests.

## Authors’ contributions

MB wrote the paper, SA collected the images, GI and MP wrote the references and the figure captions, and GG revised the paper. All authors read and approved the final manuscript.
